# Supplementary material for: The CYP80A and CYP80G Are Involved in the Biosynthesis of Benzylisoquinoline Alkaloids in the Sacred Lotus (Nelumbo nucifera)
Source: Int J Mol Sci. 2024 Jan 5;25(2):702. doi: 10.3390/ijms25020702 (PMC10815925; doi:10.3390/ijms25020702)

Supplementary Table S1. Primers for gene clone

Name	Sequence (5’ to 3’)
NnCYP80A-F	ATGGCTCTACTAGCCTTGTTTATTC
NnCYP80A -R	TTATTTTCTCAAGGTGGGAATGACGACG
NnCYP80G -F	ATGGCTCTACTAGTCTCGTTTA
NnCYP80G -R	TTACTTCCTCAACTTGGGAATGACTACC
NnCYP80B -F	ATGGAAATCATGGCTCAAGCAGC
NnCYP80B -R	TTAATAATTTCTCCTTCTCAGTTTAGG

Supplementary Table S2. Primers for CYP80G mutation

Name	Sequence (5’ to 3’)
G-39G-F	CTTTTCATGGCCAATTATAGcGACCCTGTTG
G-39G-R	CTTCAGCTTGGGCAACAGGGTCGCTATAATTGG
G-69G-F	CTAAAATTTGcGGTTGAGCCCGTTGTTGTGG
G-69G-R	GGGCTCAACCGCAAATTTTAGGAGCATTAGTG
G-A211E-F	ATGATTCGGCAGCTGATGATATTGGAAGCCATCCCAAATCT
G-A211E-R	CTTCCAATATCATCAGCTGCCGAATCATGCCCTTCATACCCAAGT
G-288P-F	GAAACGTTTGcACCTGGTTCAGACACTAGTAC
G-288P-R	TGAACCAGGTGCAAACGTTTCCAGAAGCATGG
G-425R-F	GCCGGCAGAgcAATCTGTCCAGGGTTGTCAT
G-425R-R	TGGACAGATTGCTCTGCCGGCACCAAATGGTA
G-427C-F	AGAAGAATCcgTCCAGGGTTGTCATTGGCAAC
G-427C-R	CAACCCTGGACGGATTCTTCTGCCGGCACCA

Figure S1. Sequence alignment of *CjCYP80G2* and *NnCYP80G*. The yellow rectangles is the mutation site.

```
          1        10        20        30        40        50        60
CjCY80G2  MDLQIALFSLIPVILVFILLLKPKYKNLPPGPHPWPLIGNLPILFTNTEVPLHITLANMA
NnCYP80G  MALLVSFIFFLLSILSVVLFLKPSPNNLPPGPFSWPIIG...TLLPKLKKQPHVELSKLA

                   70        80        90       100       110       120
CjCY80G2  RTHGPIMILWLGTQPTVMASTAEAAMEILKTHDRIFSARHIRMSFRLKHHIKYSLVWSDC
NnCYP80G  QRFGPLMLLKFGVEPVVVASSHVAAVEVLKNQDRLLSGRFAPHSVRIKGYIEHSMVWADC

                  130       140       150       160       170       180
CjCY80G2  TDYWKLLRKIVRTEIFSPKMLQAQSHVREQKVAELIDFLRSKEGQVVKISQFVFGTLLNI
NnCYP80G  TDYWKMVRKVWRTELFSTKMLDIQAHAREEKVSELMKFLIRKEGEKVNFADVIFGSILNI

                  190       200       210       220       230
CjCY80G2  LGNVVFSKDVFVYGDETDK.GGIQNLIREMLMIGAEPNVAEFYPSLEELDLQGLKKKCDE
NnCYP80G  LGALIFSKDVYDFEDRTDNNLGMKGMIRQLMIIAAIPNLADLYPILGGSDFQGLRKASAA

         240       250       260       270       280       290
CjCY80G2  RFIRVMKMWEGTVKERKANRNEESKDMLDVLLANDFNDAQINALFLETFGPGSETSSATI
NnCYP80G  CVKRMNESWAAIVKQRRKNDDHSKNDFLQVLLDSGFSDPQIDAMLLETFGPGSDTSTSTI

         300       310       320        330       340       350
CjCY80G2  EWVIAELIKSPKEMAKVRKELNEVVG.TSTIKESDLPQLPYLQACIKEAMRLHPAAPFLL
NnCYP80G  EWAMAELLRNPEKLVKVREELDRVIRRSNNVKESDLPNLPYLHACVKETLRLHPPVTFLL

           360       370       380       390       400       410
CjCY80G2  PRRAAETCEVMGYTIPKNSQVLVNAYAIGRDPKSWKDPSTFWPERFLESDVDFHGAHYQF
NnCYP80G  PHRAMETCQMMNYTIPKGCQLMVNTYAIGRDSKTWEKPLSFLPERFLNSELDYQGNDFQY

           420       430       440       450       460       470
CjCY80G2  IPFGSGRRTCVGMPLATRTIPLIVGSLVHNYDFGLPGGNRPEDLKMNEMLSLTLAIDPSL
NnCYP80G  IPFGAGRRICPGLSLATRVVRLILASLLHTFDWSLPDGMHPDELDMNDKFGLALQKDIPL

           480
CjCY80G2  CVVPKAR.
NnCYP80G  VVIPKLRK
```

Figure S2. The biosynthesis pathways of bisbenzylisoquinoline alkaloids and aprophine alkaloids.

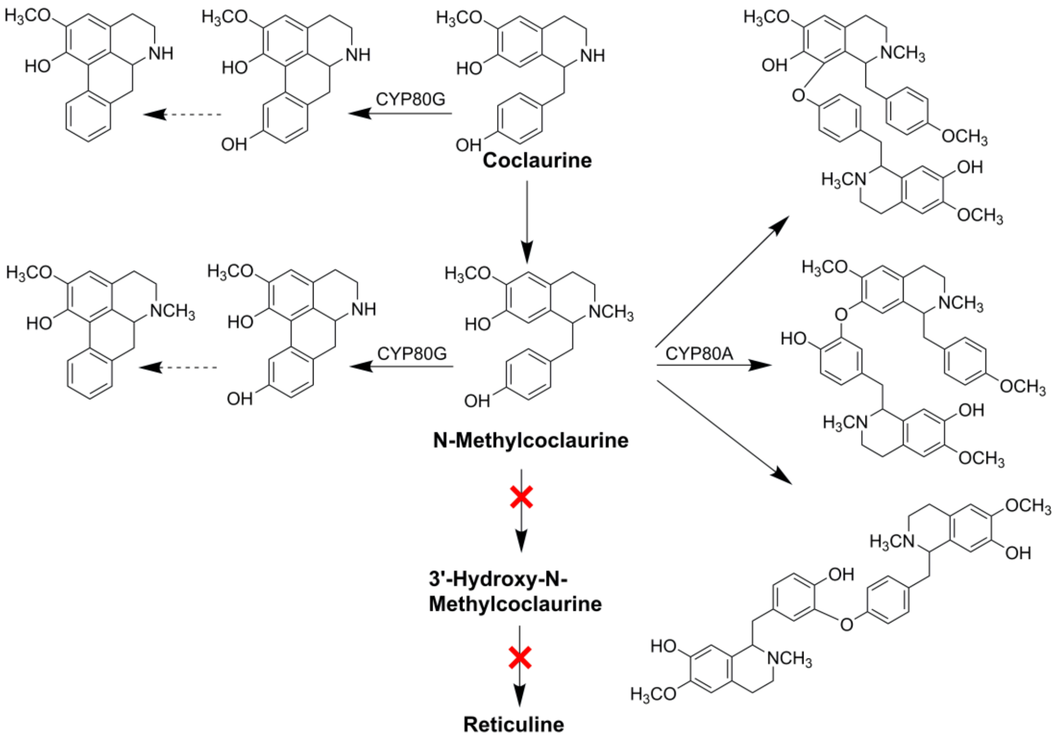

Figure S3. The circular dichroic chromatography of *(S)-N*-methylcoclaurine and *(R)-N*-methylcoclaurine.

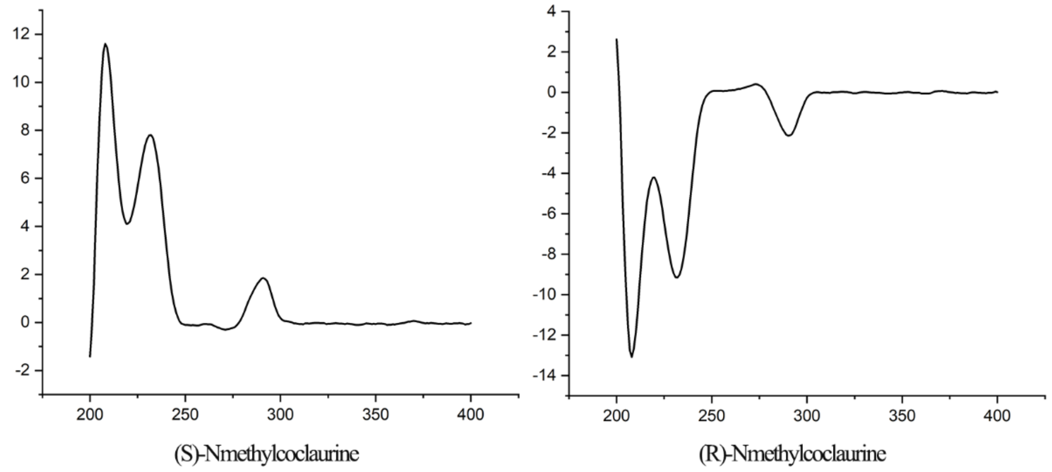

Supplement: Supplementary file 1 [file ijms-25-00702-s001.zip › ijms-2676057-supplementary.pdf]
